# Hepatoprotective Potential of *Sargassum muticum* against STZ-Induced Diabetic Liver Damage in Wistar Rats by Inhibiting Cytokines and the Apoptosis Pathway

**DOI:** 10.1155/2019/7958701

**Published:** 2019-02-27

**Authors:** Mohammed M. Safhi, Mohammad Firoz Alam, Sivagurunathan Moni Sivakumar, Tarique Anwer

**Affiliations:** ^1^Department of Pharmacology and Toxicology, College of Pharmacy, Jazan University, Jazan, Saudi Arabia; ^2^Department of Pharmaceutics, College of Pharmacy, Jazan University, Jazan, Saudi Arabia

## Abstract

Liver inflammation and necrosis are the foremost problems interlinked with diabetes mellitus (DM). The methanolic extract of *Sargassum muticum* (MESM) plays a hepatoprotective role in streptozotocin- (STZ-) induced hepatic injury. In this study, STZ exposure induced diabetes that augmented hepatic damage, which was reflected in serum enzyme markers, the cytokine network, and caspase-3 and caspase-9 levels in Group 2. Exposure to the MESM tremendously modulated the levels of hepatic enzyme markers ALP, ACP, ALT, and AST in Groups 3 and 4. The cytokine network was well regulated by suppressing the release of cytokines, and the levels of caspase-3 and caspase-9 were also reduced in Groups 3 and 4. The present study suggests that MESM treatment at 200 and 500 mg protected the liver and also minimizes the glucose level. Thus, the MESM plays a key role in rejuvenating the liver and can modulate diabetes's pathogenic effect by reducing the glucose level.

## 1. Introduction

Diabetes mellitus is a metabolic disorder that has developed into a global life-threatening disease; it is characterized by polyphagia, polydipsia, polyuria, blurred vision, and weight loss [1]. Scientific reports show that diabetes mellitus is associated with liver damage or abnormalities leading to acute liver disease [2]. The prevalence of chronic liver disease has been anticipated in the next decade due to metabolic syndrome whose symptoms include abdominal obesity as well as insulin resistance [3]. Insulin resistance is one of the vital causes of type 2 diabetes mellitus and leads to a hyperglycemic state and oxidative stress, causing liver tissue necrosis [4–6]. A healthy liver regulates its cellular growth and function by the homeostatic mechanism, conserving persistent tissue mass compared to levels of metabolic stress in the body. The liver plays a predominant role in regulating glucose homeostasis, suggesting evidence of pathogenesis of glucose intolerance in diabetes mellitus. Research studies have shown that diabetes mellitus is caused by oxidative stress that leads to the production of reactive oxygen species (ROS), which are the major causes of cellular damage [5, 7]. Therefore, diabetes mellitus is highly related to liver inflammation, cirrhosis, apoptosis, and *β*-cell dysfunction, and it ultimately causes liver malfunction. Seaweed is a potential resource of bioactive compounds, and their properties are well known across the world [8]. *Sargassum muticum* is an olive-brown alga which grows in the infralittoral zone on rocks, stones, and shells. Furthermore, *Sargassum muticum* is a very common seaweed in the Red Sea of Jazan Province, KSA, and its biological and pharmaceutical properties have not yet been explored. Thus, the present study's objective was to explore the hepatoprotective effect of the seaweed's extract and establish that the molecular mechanism is of primary importance against STZ-induced diabetic liver damage.

## 2. Materials and Methods

### 2.1. Chemicals and Reagents

Streptozotocin (STZ) and oxidative stress chemicals were procured from Sigma-Aldrich, USA through Bayoni Trading Company KSA. The assay kits such as glucose, aspartate aminotransferase (AST), alanine transaminase (ALT), alkaline phosphatase (ALP), and acid phosphatase (ACP) were procured from Crescent Diagnostics, Jeddah, Saudi Arabia. Proinflammatory cytokine assay kits (IL-1*β*, IL-2, and TNF-*α*) and apoptosis assay kits (caspase-3 and caspase-9) were purchased from Abcam, USA, through local suplier of Saudi Arabia.

### 2.2. Seaweed Collection and Extraction

*Sargassum muticum* seaweeds were collected from the Red Sea of Jazan Province of Saudi Arabia. Collected seaweeds were further cleaned and dried at room temperature under shade for two weeks. The dried seaweed samples were collected and cut into small pieces. The small pieces were powdered using a grinder, producing a fine powder. The powder sample was pooled and stored in an airtight container for further phytochemical extraction. The extraction was performed using a Soxhlet apparatus. In brief, 200 mg of seaweed powder was packed in the Soxhlet apparatus, and methanol was used as a solvent to extract the phytoconstituents by continuous hot percolation [9]. This was done by maintaining the mantle temperature at 60°C for five hours. The extract was transferred into a beaker, and the solvent was evaporated by keeping the beaker at room temperature. After drying, the extract was scraped from the beaker, pooled, and stored in an airtight container for further chemical analysis. The standardized phytochemical test was performed to find the various active constituents.

### 2.3. Animal Study Protocol

Healthy male Wistar rats weighing 150–200 g were obtained from the Central Animal Laboratories, Jazan University, Jazan. The animals were acclimatized under standard protocols: the temperature was maintained at 22 ± 0.8°C and the humidity was about 56 ± 6%, achieved by exposing with an alternating 12 h light/dark cycle. The animals were supplied with fresh water and fed with standard pellets. The experiments on the animals were carried out according to the guidelines of the Institutional Animal Ethical Committee. The experimental protocols were approved by the Institutional Animal Ethical Committee, College of Pharmacy, Jazan University, Jazan, KSA. The reference number of approvals for conducting this research work is IAEC/COP/JU/KSA 005, dated 05 Dec. 2016.

### 2.4. Induction of Diabetes Mellitus

Diabetes mellitus (type 2) was induced by injecting a single dose of streptozotocin (STZ) (60 mg/kg body weight i.p.) and nicotinamide (120 mg/kg i.p.) [10]. The glucose level was measured from the tail vein blood using a glucometer (Accu-Chek, Roche Diagnostics, Penzberg, Germany). Diabetes was also confirmed by estimation of the glucose level after 72 hrs by glucose assay kit. Those animals that represented a 200 mg/dl glucose level or more were considered diabetic rats and further divided into different groups.

### 2.5. Experimental Design

Five groups of male rats were made, and six animals were designated in each group and categorized as follows:

1. *Normal Control Group (Group 1)*. All the animals received only vehicle
2. *Diabetic (Group 2)*. All the animals received only a single dose of STZ i.p. and nicotinamide i.p.
3. *Diabetic with Treatment with the MESM (200 mg) (Group 3)*. All the animals received a single dose of STZ i.p. and were treated with 200 mg of the MESM orally once a day for seven days
4. *Diabetic with Treatment with the MESM (500 mg) (Group 4)*. All the animals received a single dose of STZ i.p. and were treated with 500 mg of the MESM orally once a day for seven days
5. *MESM (500 mg) Only (Group 5)*. This group was given 500 mg of the MESM orally once a day for seven days

At the end of the experiment, i.e., on the 8th day, blood samples were collected from all the groups of rats. The blood samples were collected in sterile glass tubes and kept in a slanting position to allow the serum to ooze out from the clotted blood. Finally, the serum was separated by centrifugation at 4000 rpm and stored at -20°C for the biochemical estimation.

### 2.6. Estimation of Glucose

The glucose level was estimated in serum with the help of a glucose assay kit (Crescent Diagnostics, Jeddah, Saudi Arabia).

### 2.7. Assessment of Serum Markers for Liver Function

#### 2.7.1. Estimation of Alkaline Phosphatase (ALP) and Acid Phosphatase (ACP)

ALP and ACP were determined as per the assay kit guideline by colorimetric detection at 405 nm using a microplate reader. In this assay, p-nitrophenyl phosphate (p-NPP) was used as a phosphatase substrate. The endpoint detection was based on the formation of a yellow color because of dephosphorylation by ALP and ACP individually. The sample concentration was calculated by extrapolating on the standard curve.

#### 2.7.2. Estimation of Alanine Transaminase (ALT)

Alanine transaminase (ALT) was determined as per the assay kit guideline by colorimetric detection at 570 nm using a microplate reader. In this assay, ALT catalyzed the amino group of *α*-ketoglutarate to glutamate and the pyruvate resulted in a colorless endpoint and was measured. The sample concentration was calculated by extrapolating on the standard curve.

#### 2.7.3. Estimation of Aspartate Aminotransferase (AST)

Aspartate aminotransferase (AST) was determined as per the assay kit guideline by colorimetric detection at 450 nm using a microplate reader. In this assay, AST catalyzed the amino group of *α*-ketoglutarate to glutamate and oxaloacetate resulted in a colorless endpoint. The sample concentration was calculated by extrapolating on the standard curve.

### 2.8. Proinflammatory Cytokine Studies

The simple step sandwich ELISA assay was used according to the company protocol to determine interleukin-1*β* (IL-1*β*), interleukin-2 (IL-2), and tumor necrosis factor-*α* (TNF-*α*) in the serum sample. The end reaction was enumerated by the concentration of color development, which was measured at 450 nm using the ELISA Reader. The sample concentration was calculated by extrapolating on the standard curve.

### 2.9. Apoptosis Marker (Caspase-3 and Caspase-9) Studies

Caspase-3 and caspase-9 were determined according to the company protocol in the serum sample by using the spectrophotometric technique. The end reaction was quantified by paranitroaniline (pNA) light emission, which was measured at 405 nm using a microplate reader. The sample concentration was calculated by extrapolating on the standard curve. Caspase-9 was determined in the serum sample by the spectrophotometric technique. The end reaction was quantified by pNA light emission after the cleavage of the substrate LEHD pNA which was measured at 405 nm using a microplate reader. The sample concentration was calculated by extrapolating on the standard curve.

### 2.10. Statistical Analysis

The statistical significance was determined using one-way analysis of variance (ANOVA) using GraphPad InStat software. The levels of significance were taken as *p* < 0.05, *p* < 0.01, and *p* < 0.001. Dunnett's multiple comparison tests were also performed, and the values are expressed as mean ± standard deviation.

## 3. Results

### 3.1. Phytochemical Analysis

The phytochemical analysis of the methanolic extract of *Sargassum muticum* (MESM) showed various phytoconstituents, such as carbohydrates, alkaloids, proteins, tannins, and glycosides (Table 1). However, carbohydrate was the predominant component when compared to the rest. In this study, there were trace levels of flavonoids, terpenoids, and steroids.

### 3.2. Effects of the MESM on Glucose Levels

The glucose level was significantly increased in the diabetic group (Group 2) when compared to normal control (Group 1). Administration of two doses of the MESM (200 and 500 mg/kg) indicated a significant decline in the glucose level in the treated group (Groups 3 and 4) as compared to the diabetic group (Group 2) While in Group 5, the glucose level was normal similar to that in the control group (Table 2).

### 3.3. Effects of the MESM on Liver Function and Serum Marker Levels

The result mentioned in Table 3 is self-explanatory and explains the study's various parameters on the serum enzyme levels after treatment with the MESM. The activities of serum enzymes AST, ALT, ALP, and ACP significantly increased in STZ-induced diabetic rats when compared to normal nontreated Group 1. Meanwhile, the level of the hepatic enzyme also decreased after treatment with the MESM even at a 200 mg dosage level. Furthermore, the hepatic enzyme level significantly decreased after treatment with 500 mg of the MESM. On the other hand, the drug treatment in the seaweed control group (Group 5) did not show any significant deviation from that in the nontreated Group 1 animals.

### 3.4. Effects of the MESM on Serum Proinflammatory Cytokine Levels

**Figure 1:**
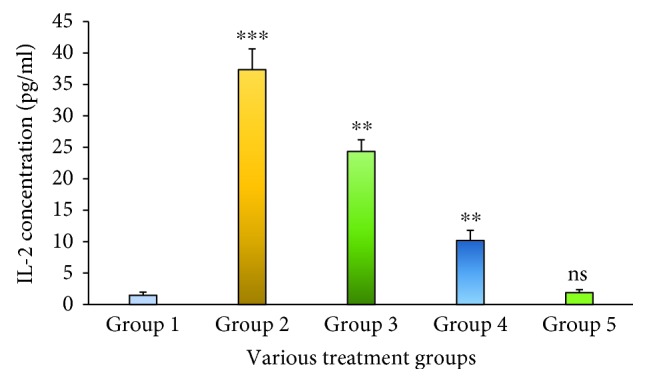
The serum IL-2 level of STZ-induced liver damage before and after treatment with the methanolic extract of *Sargassum muticum* (MESM); ^∗∗∗^*p* < 0.001, extremely significant when compared to Group 1; ^∗∗^*p* < 0.01, significant when compared to Group 1; ns: nonsignificant at *p* > 0.05 when compared to Group 1.

**Figure 2:**
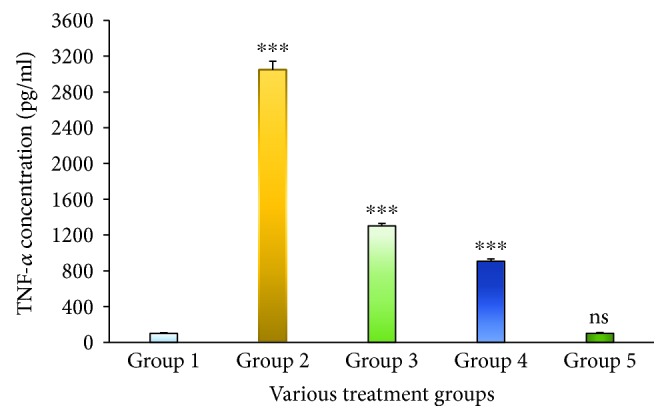
The serum TNF-*α* level of STZ-induced liver damage before and after treatment with the methanolic extract of *Sargassum muticum* (MESM); ^∗∗∗^*p* < 0.001, extremely significant when compared to Group 1; ns: nonsignificant when compared to Group 1 at *p* > 0.05.

**Figure 3:**
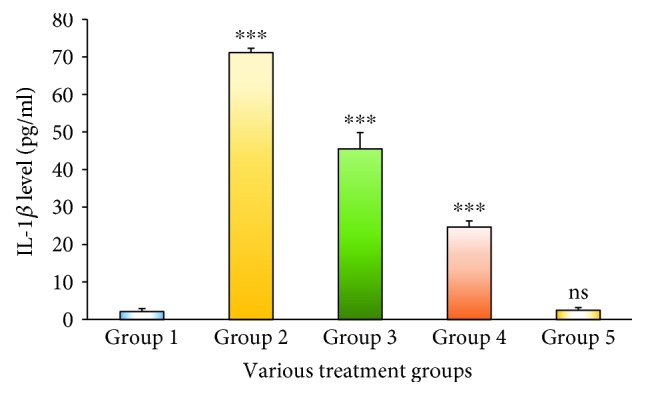
The serum IL-1*β* level of STZ-induced liver damage before and after treatment with the methanolic extract of *Sargassum muticum* (MESM); ^∗∗∗^*p* < 0.001, extremely significant when compared to Group 1; ns: nonsignificant when compared to Group 1 at *p* > 0.05.

IL-2 in the serum was quantified by an *in vitro* enzyme-linked immunosorbent assay. This proved that the Group 2 animals treated with STZ exhibited IL-2 levels which was extremely highly significant at a *p* < 0.001 level when compared to the Group 1 animals. The level of IL-2 was observed to reduce significantly at a *p* < 0.01 level when compared to that of Group 2 after treatment with the MESM, at either 200 or 500 mg (Figure 1), which was grouped into Group 3 and Group 4. Group 5 animals considered as the seaweed control study group did not elicit any significant IL-2 levels when compared to Group 1 animals. Tumor necrosis factor-*α* (TNF-*α*) is the main proinflammatory cytokine involved in the primary inflammatory mechanism. In this study, the TNF-*α* increase in Group 2 STZ-treated animals was extremely significant at a *p* < 0.001 level. The seaweed treatment (MESM), after the induction of diabetes, showed that TNF-*α* was significantly decreased at 200 and 500 mg of seaweed extracts (Figure 2). However, the comparison between Group 1 and Group 5 is nonsignificant at *p* > 0.05. IL-1*β* is a predominant proinflammatory cytokine in insulin resistance of diabetics, and it is also a key cytokine in liver damage. IL-1*β* in the serum was quantified by an *in vitro* enzyme-linked immunosorbent assay.  Figure 3 demonstrates the quantification of IL-1*β*. The study proved that the Group 2 STZ-treated animals exhibit extremely significant IL-1*β* levels at *p* < 0.001 when compared to Group 1. Group 3 and Group 4 showed a significant reduction in IL-1*β* at a *p* < 0.001 level when compared to Group 2. However, the comparison between Group 1 and Group 5 is nonsignificant at *p* > 0.05.

It is obvious from the present research findings that levels of proinflammatory cytokines IL-1*β*, IL-2, and TNF-*α* significantly increased in the STZ-induced diabetic model (Group 2) when compared to the nondiabetic model of animals (Group 1). Therefore, Group 5 was designated as the seaweed control group for establishing the MESM's toxic properties. In this study, treatment with the MESM at either 200 or 500 mg markedly inhibited the elevated cytokine levels.

### 3.5. Effects of the MESM on the Serum Apoptosis Marker Level

**Figure 4:**
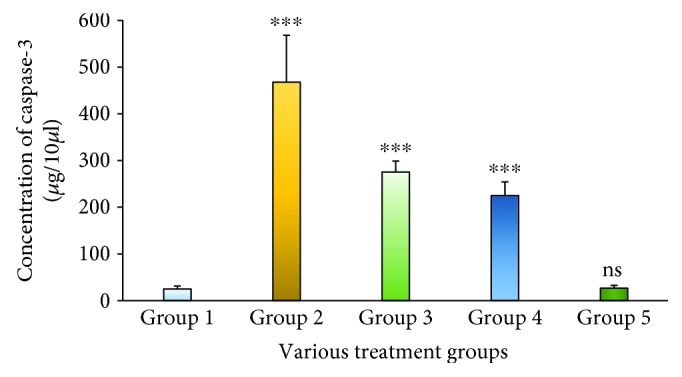
The serum caspase-3 level of STZ-induced liver damage before and after treatment with the methanolic extract of *Sargassum muticum* (MESM); ^∗∗∗^*p* < 0.001, extremely significant when compared to Group 1; ns: nonsignificant when compared to Group 1 at *p* > 0.05.

**Figure 5:**
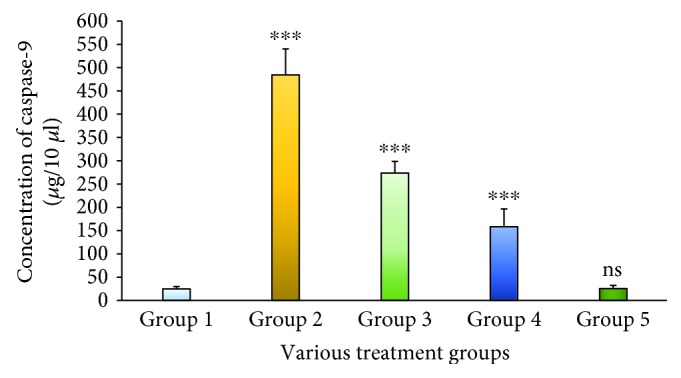
The serum caspase-9 level of STZ-induced liver damage before and after treatment with the methanolic extract of *Sargassum muticum* (MESM); ^∗∗∗^*p* < 0.001, extremely significant when compared to Group 1; ns: nonsignificant when compared to Group 1 at *p* > 0.05.

Caspase-3 is a key protein involved in the apoptosis mechanism that cleaves many cellular proteins and thus initiates the elimination of aged, damaged, and autoreactive cells.  Figure 4 exemplifies the release of protease caspase-3 against various treatment groups. The study indicates that introducing STZ led to a massive release of caspase-3 when compared to Group 1; it is an indicator of cell damage and cellular death. However, the caspase-3 level was reduced after treatment with the MESM in the 200 and 500 mg dosage forms. Fascinatingly, there is no significant release of caspase-3 in Group 5 animals that received 500 mg of the MESM twice a day. Caspase-9 is a protease enzyme involved in the apoptosis mechanism and linked with mitochondrial damage. The production of caspase-9 against various treatment groups is depicted in Figure 5. Results showed that introducing STZ in a group of animals (Group 2) led to a considerable amount of caspase-9 that indicates cellular damage. However, treatment with the MESM at 200 and 500 mg dosage levels showed a prompt reduction in caspase-9 concentration levels.

## 4. Discussion

The liver is the master organ for carbohydrate metabolic homeostasis and releases glucose according to metabolic needs. Recently, liver damage has been documented as a major complication of diabetes mellitus. Indeed, various studies suggest that mortality due to liver disease in diabetic patients is very high, even higher than cardiovascular diseases [11]. Though several pathways have been identified for liver damage, insulin resistance is the main cause of liver damage because of oxidative stress and increased production of ROS [12]. Insulin resistance causes severe hyperglycemia and does not detect the available glucose; instead, it causes the collective action of increasing the secretion of glucose-6-phosphate dehydrogenase, hexokinase, and glucokinase. The spectrum of typically occurring biochemical changes in diabetes mellitus resembles the end-stage of liver failure [13]. The liver also maintains the glucose homeostasis by encoding the genes of secretory protein hepatokines and activating either a positive or negative feedback mechanism to govern the metabolism process of the cell [14]. The potential of seaweed as a biomedicine is well known to the scientific community, and its pharmacological properties have been well documented by various researchers [14]. The work is aimed at understanding the efficacy of the MESM against STZ-induced diabetes mellitus that induced liver damage. Thus, the present study focused on the efficacy of the methanolic extracts of seaweed against various proinflammatory cytokines, serum enzymes, and apoptotic markers in liver dysfunction because of STZ-induced diabetes mellitus. Therefore, establishing biomolecular tools is of more importance for understanding the disease process, which is the prime objective for therapeutic utility. STZ is a potent chemical commonly used for the induction of diabetes. In this study, STZ-treated rats significantly developed diabetes, thereby producing hepatotoxicity as an interlinked mechanism, which is visualized as elevated levels of ALP, ACP, ALT, and AST. Ghosh et al. [7] reported that the serum enzyme levels increased in STZ-treated animals. The study was performed in dose-dependent effects from curcumin treatment for an eight-week period that exhibited beneficial effects against STZ-induced hepatotoxicity. Interleukin-2 (IL-2) is a protein produced by CD4^+^ (Th_2_) cells, to a lesser extent by CD8^+^ (Tc) cells, natural killer (Nk) cells, and natural killer T (NkT) cells. IL-2 is a cytokine that plays a dual role having proinflammatory and anti-inflammatory effector functions. Interleukin-2 (IL-2) molecule signals influence many lymphocytes during proliferation; the differentiation leads to an immune response and maintains homeostasis [15]. Studies suggest that IL-2 signals affect the action of CD8+ cells in mounting immune responses, and therefore, the action as an inflammatory response is quite limited [16, 17]. IL-2 utilizes the actions via the IL-2R*α*, IL-2 R*β*, and IL-2R*γ* receptor-mediated JAK/STAT pathway. An earlier report suggested that soluble IL-2R was involved in activating immune cells in various inflammatory disease conditions [18]; elevated soluble IL-2R has been described for diverse hepatic disorders [19]. The present study suggests that the IL-2 level increased in the group of animals that received STZ, confirming the induction of hepatic injury which was also reflected in the various enzyme parameter studies. However, as done in enzyme studies, the level of IL-2 has been reduced after treatment with the MESM at either 200 or 500 mg. On the other hand, the seaweed control group of animals only received the MESM without any STZ induction, demonstrating the nonsignificant deviation from the normal untreated Group 1. From the toxicity study, it is apparent that the MESM did not show any adverse effects and appealing nontoxic properties.

Tumor necrosis factor-*α* (TNF-*α*) is a protein involved in inflammatory reactions and closely associated with apoptosis [20, 21]. TNF-*α* is mostly derived from macrophages, which have been reported to promote insulin resistance [22]. TNF-*α* and NF-*κ*B are used as inflammatory biomarkers [23], which arise in various acute and chronic liver diseases and are believed to be involved in liver damage and repair processes. TNF-*α* has a role in regulating apoptosis and inflammatory processes in diabetes and hepatic injury [24]. Compared with the earlier report, the present study showed an increased TNF-*α* level proportionately and extremely significant at a *p* < 0.001 level. The results indicated that the induction of insulin resistance and hepatic damage occurred because of STZ treatment. It is very interesting to note that after the treatment with the MESM, at either 200 or 500 mg, the hepatic inflammation reduced, which was reflected in the TNF-*α* level in the serum. Earlier studies suggested that TNF-*α*-mediated inflammatory responses were enhanced by IL-1*β* [25]. IL-1*β* is a predominant cytokine in inflammatory conditions, especially in diabetic mellitus. IL-1*β* is synthesized as an inactive precursor protein pro-IL-1*β* which is metabolized by the enzyme caspase-1 to give active IL-1*β*. Earlier reports showed that IL-1*β* is associated with defective secretion of insulin as well as the development of insulin resistance [26–28]. In this study, IL-1*β* levels increased significantly in the STZ-induced group of animals when compared to the normal Group 1, which represented the induction of diabetes and hepatic injury [29]. On the other hand, the acute toxicity group showed no significant increase in IL-1*β* when compared to the normal control. Treatment with the MESM, at either 200 or 500 mg, significantly reduced IL-1*β*, proving the efficacy of the MESM against hepatic injury in the STZ-induced diabetic animal model. Though the proinflammatory cytokine levels were well elevated, TNF-*α* exhibited a pivotal role of inducing apoptosis through a classical intermolecular cascade pathway leading to cellular death. Caspases are a group of proteolytic enzymes responsible for cellular damage. Caspases can be classified into two pathways: the proapoptotic and proinflammatory subfamilies. In this study, the apoptosis was confirmed by increased caspase-3 and caspase-9 levels due to STZ induction [30]. Thus, it is proved that STZ induction leads to hepatic cell damage and cell death. The results indicated that the MESM exhibited anti-inflammatory properties probably because of the polysaccharide proteins and the presence of trace amounts of steroids. Furthermore, hepatoprotective properties might also be influenced by the presence of trace flavonoids. Earlier reports also showed that the polysaccharides from seaweeds exhibited anti-inflammatory and antidiabetic activities (McIlwain et al. [31]).

## 5. Conclusion

Liver damage is one of the foremost complications in association with diabetes mellitus, especially type 2 diabetes mellitus. The pathological consequence of liver damage is characterized by the elevation of liver enzymes. However, the molecular background of the disease is appealing the elevation of proinflammatory cytokines and the caspase system. There has been a global challenge of interest in drugs from natural origin, especially from an herbal origin in the recent past. Though herbal medicines are effective in the treatment of various ailments, especially in liver damage, their efficacy is still unclear because of being nonscientifically exploited. Recently, the biomedical importance of seaweed has been focused to develop novel biomedicines, and, in this work, the hepatoprotective effect of the seaweed *Sargassum muticum* extract has been demonstrated scientifically. Thus, the methanolic extract of seaweed has hepatoprotective effects by expressing the modulation of various cytokines and liver enzyme parameters. Linear trends were observed at *p* < 0.001 between the groups treated either at a 200 mg or at a 500 mg level for all the serum enzymes as well cytokine networks and the caspase system that have been screened in this work. Moreover, the extract was not inducing any toxic effect in the treated animals. Thus, the *Sargassum muticum* extract plays a key role in rejuvenating the liver cell and also minimizing the glucose level that can modulate the diabetic pathogenic effect which is important for therapeutic practice.

## Figures and Tables

**Table 1:** Phytochemical constituents of *Sargassum muticum*.

Active constituents	Result
Carbohydrate	+++
Proteins	++
Alkaloids	++
Tannins	++
Glycosides	++
Flavonoids	+
Terpenoids	+
Steroids	+

+++: predominantly present; ++: moderately present; +: present in trace level.

**Table 2:** Effect of the methanolic extract of *Sargassum muticum* (MESM) on serum glucose levels.

Different groups	Glucose (mg/dl) (mean ± S.D)#
Group 1	95.41 ± 2.8
Group 2	352.52±8.5^∗∗∗^
Group 3	180.30 ± 14.3^∗^
Group 4	127.16±9.4^∗∗^
Group 5	97.32 ± 3.9^ns^

Group 1: normal control; group 2: STZ induction group; Group 3: treatment with a lower dosage (200 mg/kg) of the MESM after induction of STZ; Group 4: treatment with higher doses (500 mg/kg) of the MESM after induction of STZ; Group 5: seaweed control studies. ^#^Each value is mean ± S.D. for six rats in each group (*n* = 6); ^∗∗∗^*p* < 0.001, extremely significant; ^∗∗^*p* < 0.01, significant; ^∗^*p* < 0.05, significant when compared to NC; ns: nonsignificant (*p* > 0.05).

**Table 3:** Effect of the methanolic extract of *Sargassum muticum* (MESM) on liver function markers in diabetic rats.

Treatment groups	ALP (U/l)	ACP (U/l)	ALT (U/l)	AST (U/l)
Group 1	45 ± 2.8	11 ± 0.9	59.5 ± 3.7	36.33 ± 1.5
Group 2	387.5±28.5^∗∗∗^	27.16±1.3^∗∗∗^	119±4.1^∗∗∗^	85±2.5^∗∗∗^
Group 3	186.3±5.6^∗∗∗^	16.33±1.0^∗∗∗^	94.83±3.6^∗∗∗^	57.16±1.2^∗∗∗^
Group 4	77.16±5.7^∗∗∗^	13.5±1.4^∗∗^	75.33±2.3^∗∗∗^	40±2^∗∗^
Group 5	44.3 ± 3.9^ns^	11.16 ± 0.8^ns^	60 ± 3.1^ns^	37 ± 1^ns^

Group 1: normal control; Group 2: diabetic rat; Group 3: diabetic rat plus treatment with a lower dosage of the MESM; Group 4: diabetic rat plus treatment with a higher dosage of the MESM; Group 5: seaweed control studies. Each value is mean ± S.D. for six rats in each group (*n* = 6); ^∗∗∗^*p* < 0.001, extremely significant; ^∗∗^*p* < 0.01, significant when compared to NC; ns: nonsignificant (*p* > 0.05).

## Data Availability

The data used to support the findings of this study are included within the article.
